# Myofilamental integrity of the myometrium due to cold ischaemia stress during autotransplantation in the experimental sheep model

**DOI:** 10.1371/journal.pone.0338477

**Published:** 2025-12-09

**Authors:** Bálint Farkas, Katalin Türmer, Martin Rozanovic, Kálmán Kovács, József Bódis, Gábor Jancsó, Gábor Fazekas, Dávid Szatmári

**Affiliations:** 1 Department of Obstetrics and Gynecology, University of Pécs, Pécs, Hungary; 2 National Laboratory on Human Reproduction, University of Pécs, Pécs, Hungary; 3 Department of Biophysics, Medical School, University of Pécs, Pécs, Hungary; 4 Department of Anaesthesiology and Intensive Therapy, University of Pécs, Pécs, Hungary; 5 Department of Vascular Surgery, University of Pécs, Pécs, Hungary; IIIT Kurnool: Indian Institute of Information Technology Design and Manufacturing Kurnool, INDIA

## Abstract

Uterine transplantation is currently the only solution that enables women with absolute uterine factor infertility to become pregnant and give birth to a child. In the preparatory phase of a human uterus transplantation, the sheep is the most recommended species. Cold ischaemia, i.e., a period of reduced or absent blood flow at cold conditions, can significantly impair the function of the transplanted organ. Cold ischaemia impairs smooth muscle function in general and reduces smooth muscle contractile activity. However, it seems to provide some protection against cold storage. Our main goal was to investigate the molecular mechanisms leading to reversible changes in myometrial myofilaments and to distinguish these from permanent changes, which was supported by histological imaging of uterine samples. Using fluorescence spectroscopy, we investigated important interactions between major components of smooth muscle such as actin and tissue-specific actin-binding proteins. We characterized functional changes by denaturation sensitivity and protein-protein interactions under low and high salt conditions by intrinsic tryptophan, Alexa488-phalloidin and eosin fluorescence emission spectroscopy assays. Our results suggest that short-term cold ischaemia causes minor disruption of muscle cells. The protein extracts of myometrium contained large amounts of actin, which was present in soluble complexes with actin-binding proteins after ischaemic stress. The results indicate that the contractile filament system underwent molecular stabilization and reassembly due to ischaemic stress and that the actin monomers were unable to form polymers due to increased heterologous protein-protein interactions. The content of necrotic proteins cannot be detected after brief ischaemia, but eosin selectively binds to large proteins (caldesmon, myosin chains, tropomyosin) and protein complexes. Based on these results, we can assume that short-term preservation of cold ischaemia in uterine transplantation reduces the risk of using it in clinical trials for complete myometrial recovery after reperfusion.

## Introduction

Uterus transplantation (UTx) is a novel clinical method [1] which enables women with absolute uterine factor infertility (AUFI) to bear a child and give birth. It is a rare condition that affects an estimated 3–5% of women worldwide [2,3]. In their case, infertility occurs due to the uterus is either congenitally missing (Müllerian agenesis) or had been removed because of necessity (benign or malignant gynecological diseases), or not functioning and therefore not suitable for pregnancy. The first human uterus transplantation with living donation (LD) was performed in 2000. Interestingly, the procedure was technically successful, but the transplanted organ had to be removed three months later due to rejection [4]. Nevertheless, this early experiment emphasized the importance of UTx and led to the first successful LD UTx by Brännström and his colleagues in February 2013, which resulted in the birth of the first child after UTx in September 2014 [5,6]. In order to initiate a human UTx clinical trial, our group has performed UTx operations on both human cadavers and on large animals [7,8].

Surgical success in UTx can be defined by the immediate and complete restoration of blood flow to the transplanted organ, which is the key to avoid long-term rejection. The aim of the surgery is the functional restoration of uterine function and thus the achievement of fertility. Among the numerous animal species [8–12], the most recommended for the preparation of human uterine transplantation is the sheep (*Ovis aries*), as the size of its uterus and the characteristics of its supply vessels, including the dimensions of the feeding arteries and veins are very similar to human [7,13–19]. Both non-rejectable autologous and rejectable allogeneic UTx have been tested in sheep models [20,21]. Autotransplantation does not require immunosuppression and natural mating is also possible, resulting in a 75% conception rate [20,22]. This was the main reason why we chose the sheep model for our UTx investigations.

As it was revealed previously, cold ischaemia, i.e., a period of reduced or absent blood flow at cold temperatures, can significantly impair the function of the transplanted organ through thermal denaturation analysis of the tissue [23]. Most available data are from rodent models and suggest that 24 h cold ischaemia does not lead to irreversible morphological changes in the uterus, but 48 h preservation leads to tissue necrosis after transplantation [24–26]. An earlier trial in sheep showed that the uterus tolerates prolonged cold storage prior to transplantation well. After 24 hours of ischaemic cold preservation, viable uteri were ascertained by macroscopic and histological examination after autotransplantation [27]. In a sheep model, normal contractility and normalized lactate levels were measured after one hour of cold ischaemia and subsequent reperfusion [24,28]. The optimal tolerable duration of cold ischaemia in the human uterus has not yet been clearly established. However, the tolerance of human myometrial tissue to cold ischaemia is at least 6 hours in a suitable preservative solution [24,29].

Cold ischaemia can significantly impair smooth muscle function [30]. In the context of organ transplantation, prolonged cold ischaemia may impair smooth muscle cell recovery [31] and accelerate the development of allograft vasculopathy. Ischaemia generally reduces the contractile activity of vascular smooth muscle [32,33]. However, it appears to protect smooth muscle from cold storage to some degree [34]. Previous publications have found that short-term (less than 6 hours) ex vivo cold storage of smooth muscle samples does not impair their function [35–37]. However, long-term (more than 6 hours) cold ischaemia reduces the solubility of actin filaments and interfering cytoskeletal proteins in endothelial cells [38,39] and causes reversible cytoskeletal changes associated with significant gaps in the endothelial monolayer and activation of inflammatory signaling pathways [39]. Against the background of ischaemic stress, Ca^2+^ influx restores the actin monomer pool [39,40] and improves cell survival with the help of actin-binding proteins (ABPs) [41–46].

Since there is no model that accurately represents the molecular events during short-term cold ischaemia in the context of uterine transplantation, it may be a valuable goal to investigate the molecular mechanisms that lead to reversible changes in myometrial myofilaments and to distinguish these from permanent changes. Using fluorescence spectroscopy, we investigated important interactions between key components of smooth muscle such as actin [47] and tissue-specific ABPs such as tropomyosin, caldesmon, calponin and specific myosin light chains [48]. Smooth muscle extract contains a wide range of proteins with tryptophan residues. *Ovis aries* smooth muscle actin contains 4, myosin light chain kinases 33, myosin heavy chains 19, calponin 2, caldesmon 4, desmin 1 and actinin 23 tryptophan residues, whereas tropomyosin does not contain tryptophan (https://www.ncbi.nlm.nih.gov/). Therefore, the study of fluorescence emission-coupled structural dynamics of protein chains can be performed based on these intrinsic probes. Due to the relatively high number of tryptophan residues in smooth muscle proteins, we can assume that all of them are localized on less or highly flexible chains or nearby of binding sites [49–52]. It can be assumed that the microenvironment of tryptophan residues is sensitive to the structural dynamics of the protein and structural changes. Therefore, the intrinsic tryptophan emission spectra are sensitive to salt conditions and protein-protein interactions. If they exposed more strongly from protein structure, their emission intensity increases, and the maxima are red-shifted [53]; if they are more buried, the opposite is the case. The different ABPs can form protein complexes with actin and thereby alter their rotational diffusion, which can be measured due to the steady-state fluorescence anisotropy of the intrinsic tryptophan [54]. Phalloidin can only bind to short actin polymers. The ability of ABPs to alter the monomer-to-polymer ratio by dissociation or association of monomers can be indicated by the emission change of fluorophore-labelled phalloidin. Many types of ABPs can alter actin polymerization and thus inhibit phalloidin binding [55–60]. In addition, the fluorescence emission of eosin indicates the native dynamics of proteins, as their hydrate envelope affects eosin binding depending on surface charges [61], so that the cation binding sites of proteins contribute more strongly to eosin binding [62]. Furthermore, when comparing matured and freshly expressed proteins, eosin preferentially binds to long matured protein chains, which alters their emission spectra and indicates the ratio between fresh and matured proteins. As in the description of the ageing of smooth muscle myofilaments by cold ischaemia [63,64]. The interpretation of the molecular changes was supported by histological imaging of uterine tissue samples and the description of functional changes were investigated due to denaturation sensitivity and protein-protein interactions under low and high salt conditions.

## Results and discussion

### Morphology of the post-ischaemic myometrium

**Fig 1:**
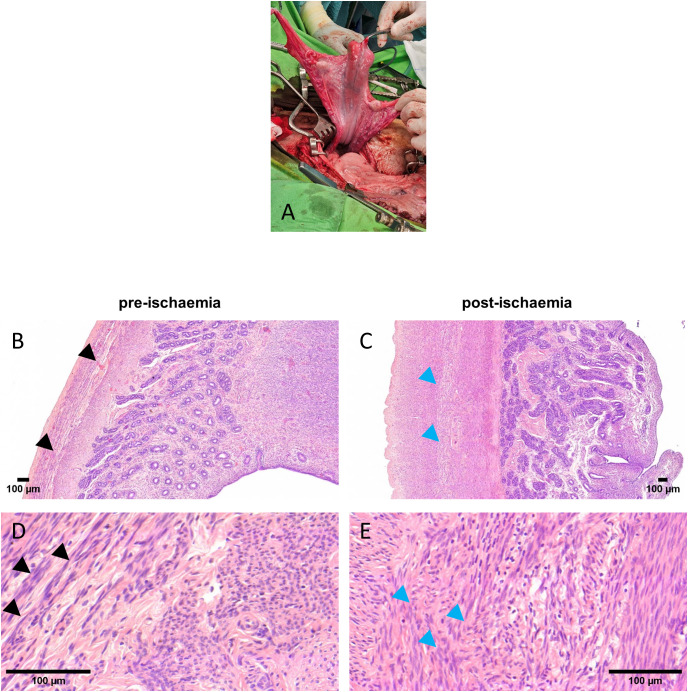
Surgery and histology of the sheep uterus before and after cold ischaemia. **(A)** Intraoperative image of the double-horned sheep uterus. **(B–E)** Bright field image of the HE-stained uterine wall, (B,D) tissues of PreI samples (B) at low and (D) at high magnification; (C,E) tissues of PostI samples (C) at low and (E) at high magnification. Black arrows show functionally organized muscle cells and blue arrows show distributed cells.

We performed cold ischaemic injuries during nine operations. For routine histological examination, hematoxylin-eosin (HE) staining was performed comparing two types of specimens: pre-ischaemia (PreI) and post-cold ischaemia (PostI) samples with the freshly harvested native uterine wall. The myometrium of the normal native uterus consists of muscle fibrils and small vessels. The PreI muscle cells are arranged in functional contractile rings and fibrils. A minor characteristic change was observed in the PostI specimens, namely the partial disruption of the fibrils and the disorganization of the smooth muscle cells (Fig 1). In addition, eosin staining was 10–20% more effective in the PostI samples. The loss of structural integrity might be related to necrotic and stress-induced changes in the smooth muscle filament system after cold ischaemia. All in all, our histological samples showed only insignificant irregularities in the muscle fibers. However, the duration of ischaemia is an important factor in maintaining structural integrity, which did not exceed the critical duration of 6 hours [30,65].

### The denaturation sensitivity of cold ischaemic muscle cell lysate

**Fig 2:**
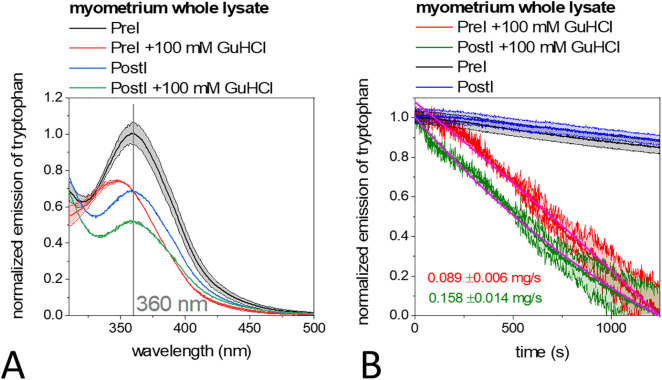
Intrinsic tryptophan fluorescence spectroscopy of whole myometrial cell lysate. **(A)** Intrinsic tryptophan fluorescence emission spectra of PreI (black and red lines) and PostI (blue and green lines) whole myometrial lysates in response to the addition of 100 mM GuHCl. **(B)** Time-dependent emission of tryptophan in the PreI (black line) or PostI lysate (blue line) in the absence and the PreI (red line) or PostI lysate (green line) in the presence of 100 mM GuHCl. Data are given as mean ± SD of five measurements on samples from nine independent animals.

To investigate the functional changes of myofilaments due to ischaemic cold injury, we performed intrinsic tryptophan fluorescence spectroscopy assays (excited at 280 nm) using whole myometrial cell lysate from uterine tissue blocks of pre- (PreI) and post-ischaemic cases (PostI). Guanidine-HCl (GuHCl) is a commonly used denaturant [66] that alters the dynamics of the hydrate envelope of proteins, which can be described by spectral changes in intrinsic tryptophan emission. The addition of 100 mM GuHCl significantly (p values < 0.05) reduced the emission of tryptophan by 25% and caused a blue shift of the emission maxima from 360 nm to 345 nm in the PreI samples (Fig 2A). The blue shift of the maxima is a common response of tryptophan that have been buried in the hydrophobic protein structure after the treatment [67,68]. In the case of the PostI samples, the emission intensity was 70% of the emission of the PreI samples. Then the tryptophan emission was significantly (p values < 0.05) reduced by 20% in the presence of GuHCl with a maximum at 360 nm (Fig 2A). This suggests that the tryptophan in the PostI samples did not respond to treatment-related structural differences. The time-dependent intrinsic tryptophan emission decreased 1.8-fold faster by GuHCl in the PostI samples than in the PreI samples (0.158 ± 0.014 mg/s and 0.089 ± 0.006 mg/s, respectively) (Fig 2B), indicating rapid denaturation kinetics of the PostI lysate, which we can interpret by less stable structural cell compartments of the PostI lysate. Here we can claim to have studied some of the processes in muscle cell compartments of the myometrium that occur during brief (1 hour) cold ischaemia in uterine transplantation. The PreI lysate samples may contain more intact cytoplasmic structures, making them more sensitive to GuHCl, and they responded with conformational changes to the denaturing treatment. The tryptophan of the PostI proteins appear to be less exposed and could be packaged in hydrophobic clefts due to the formation of protein complexes that has already taken place and thus be less exposed to environmental factors, so that their hydrate envelope is rapidly altered by GuHCl treatment [69].

### Altered protein-protein interactions due to cold ischaemia

**Fig 3:**
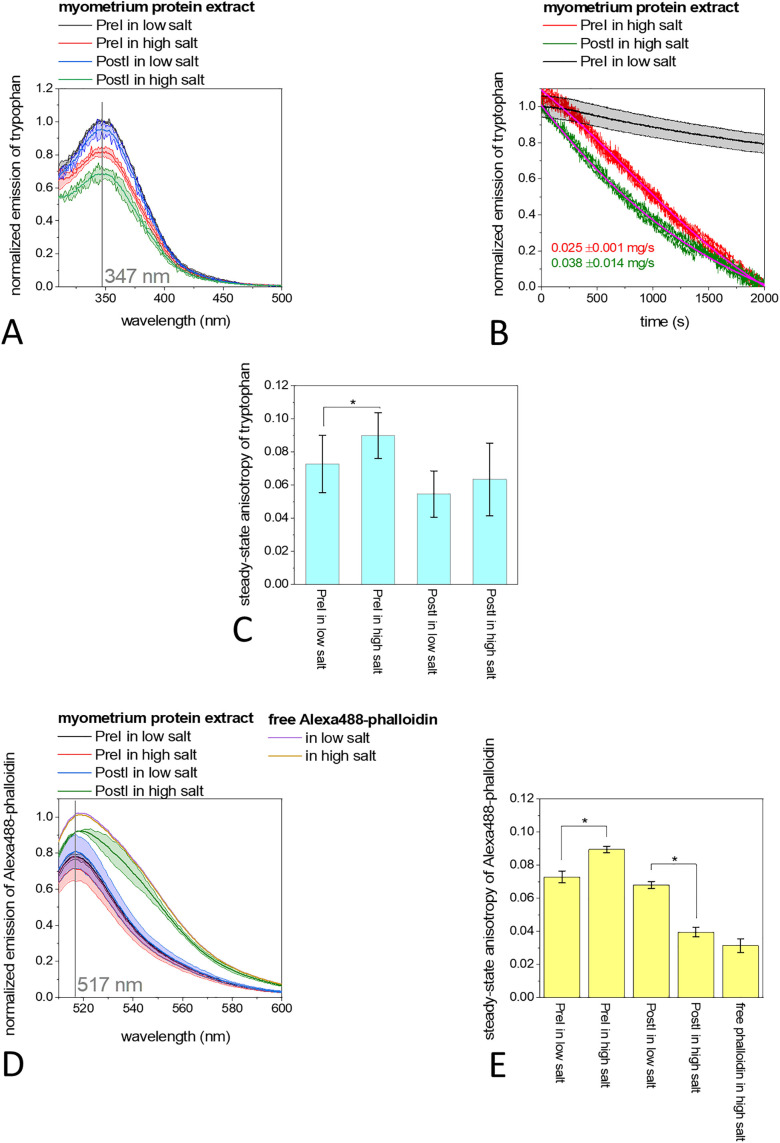
Fluorescence spectroscopic analysis of the protein extracts of myometrium. **(A)** Fluorescence emission spectra of intrinsic tryptophan in PreI (black and red lines) and PostI samples (blue and green lines) under low-salt (0 mM KCl, 0 mM MgCl_2_, 0.1 mM CaCl_2_) and high-salt conditions (100 mM KCl, 2 mM MgCl_2_, 0.1 mM CaCl_2_). All spectra had maxima at 347 nm. **(B)** Time-dependent changes of tryptophan emission in the case of PreI under low (black line) and high salt conditions (red line) and in the case of PostI samples under high salt conditions (green line). **(C)** Fluorescence anisotropy of tryptophan in PreI and PostI samples under high and low salt conditions. **(D)** Fluorescence emission of Alexa488-phalloidin under low and high salt conditions and in the absence (purple and brown lines) or presence of PreI (black and red lines) or PostI proteins (blue and green lines). **(E)** Fluorescence anisotropy of Alexa488-phalloidin under low and high salt conditions in the presence of PreI or PostI samples, using the anisotropy of free Alexa488-phalloidin as a control. Data are given as mean ± SD of five measurements on samples from nine independent animals. Asterisk indicates statistically significant differences between groups. Significance was defined as p values < 0.05.

The whole cell lysate contains membrane and cytoplasmic aggregates that was separated by ultracentrifugation to obtain a protein extract [70] (S1 Fig). Changing conditions from a low salt concentration (0 mM KCl, 0 mM MgCl_2_, 0.1 mM CaCl_2_) to a physiologically more relevant high salt concentration [71](100 mM KCl, 2 mM MgCl_2_, 0.1 mM CaCl_2_) can induce the interaction of functionally intact soluble proteins for binding or polymerization [72,73]. Regardless of the salt conditions, intrinsic tryptophan emission had maxima at 347 nm in both cases, i.e., in the PreI and PostI samples (Fig 3A), indicating that all tryptophan residues in the protein extracts are more strongly embedded in hydrophobic structures than in the case of the cell lysates. The addition of high salt significantly (p-values < 0.05) decreased the emission by 20% for the PreI and by 30% for the PostI samples, possibly due to the quenching effect of the complex formation, with the initial 5% lower emission of the PostI compared to the PreI samples. Furthermore, there was no spectral shift. The process of protein-protein binding or polymerization possibly triggered by the high salt addition. It was 1.5 times faster in the PostI samples than in the PreI samples (Fig 3B). Steady-state anisotropy can be reduced by strong light scattering in an inhomogeneous samples and increases with the size of the protein complexes [74]. The size of the protein complexes indicated by the anisotropy was significantly (p values < 0.05) increased only in the case of the PreI samples due to the high salt concentration (Fig 3C). To find out which fraction consisting free actin monomers or oligomers in the protein extracts we carried out Alexa488-phalloidin fluorescence emission assays (excited at 488 nm). Under high salt conditions, the free actin monomers can form filaments, whereby phalloidin prefers filaments among the monomers and stabilizes them by a strong interactions [75]. We observed that the emission of Alexa488-phalloidin was reduced after actin binding, with maxima shifting from 520 nm to 517 nm (Fig 3D), indicating their reduced freedom after protein binding. The quantum yield of Alexa488-phalloidin may depend on the local charges and chemical conditions as well as the altered emission polarity upon binding to actin, making it less sensitive to molecular size differences and therefore less suitable to follow actin polymerization [73,76,77]. Only in the case of the PostI samples under high salt conditions does the emission spectrum show a similar shape to free Alexa488-phalloidin (Fig 3D) with a reduced anisotropy (from 0.07 to 0.035) (Fig 3E), indicating faster rotational diffusion of phalloidin, suggesting that the phalloidin was released from proteins.

**Fig 4:**
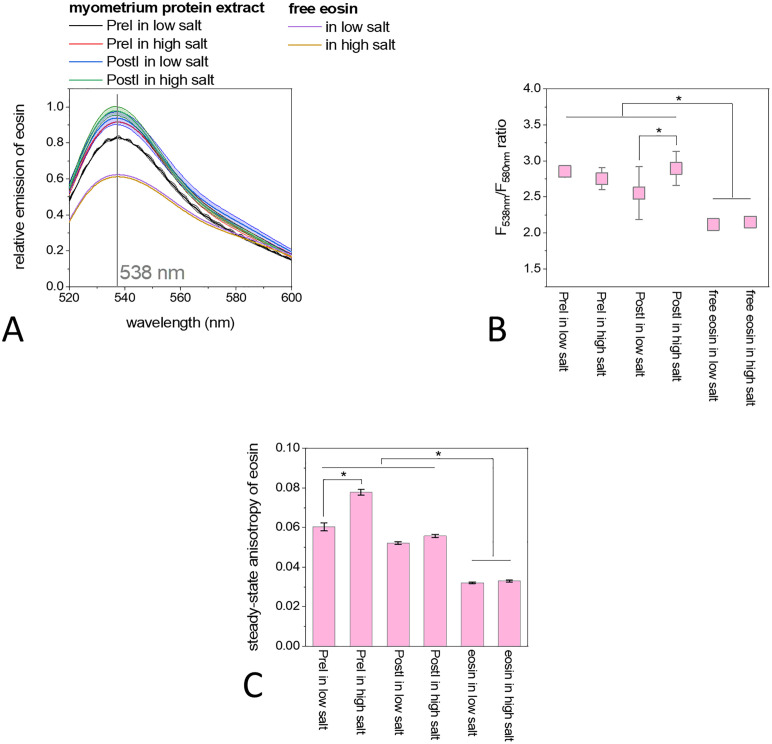
Fluorescence spectroscopic analysis of eosin-labeled myometrial protein extracts. **(A)** Fluorescence emission spectra of eosin in PreI (black and red lines) and PostI samples (blue and green lines) under low (0 mM KCl, 0 mM MgCl_2_, 0.1 mM CaCl_2_) and high salt conditions (100 mM KCl, 2 mM MgCl_2_, 0.1 mM CaCl_2_). All spectra had maxima at 538 nm. **(B)** Diagram comparing the ratio between eosin emission at 538 nm and 580 nm in different cases. **(C)** Bar graph of the steady-state fluorescence anisotropy values of eosin in different cases. Data are given as mean ± SD of five measurements on samples from nine independent animals. Asterisk indicates statistically significant differences between groups. Significance was defined as p-value < 0.05.

In order to reveal the content of necrotic proteins in the myometrial extract, we carried out eosin fluorescence spectroscopy (excited at 468 nm), in which the eosin emission is mainly green (530–570 nm) when the eosin binds to intact proteins, but shifts to the orange range (580–590 nm) due to tissue necrosis and based on histological examination [78–80]. Here, the emission of eosin had the same maxima at 538 nm in all cases, both in the PreI and PostI samples under high or low salt conditions, identical to free eosin (Fig 4A). The emission of eosin was 20% lower in the absence of proteins, suggesting that eosin emission increases due to protein binding. In the case of the PreI sample, the emission was 10% higher under high than low salt conditions. However, for the PostI samples, the emission was 13% higher at low salt conditions than for the PreI samples and did not change significantly due to the high salt addition. The high salt content possibly favors the interaction of intact PreI proteins and the binding of eosin to heterogeneous protein complexes. The ratio of emission intensities at 538 nm and 580 nm may indicate the relative amount of intact to necrotic proteins [78]. This ratio was between 2.5 and 3 in all cases, which is significantly higher (p < 0.05) than in the case of free eosin. The 538 nm/580 nm ratio was only 2.1 in the case of free eosin, indicating that the peak at 538 nm increases more than the peak at 580 nm due to protein binding. The steady-state anisotropy of eosin varied between 0.03 and 0.08 as a combined effect of the light scattering and protein binding. The anisotropy of free eosin was 0.03, while only in the case of PreI it was increased significantly (p-values < 0.05) from 0.06 to 0.079 by the high salt addition. In the case of PostI, the value was around 0.06 regardless of the salt conditions. The data show increased anisotropy of eosin in the case of PreI extracts, indicating increased binding of eosin to proteins in the presence of high salt. However, in the case of the PostI samples, the anisotropy values are at a moderate level between the PreI and free eosin values, indicating the summed effect of slow rotational diffusion and strong light scattering due to the large filament structures.

**Fig 5:**
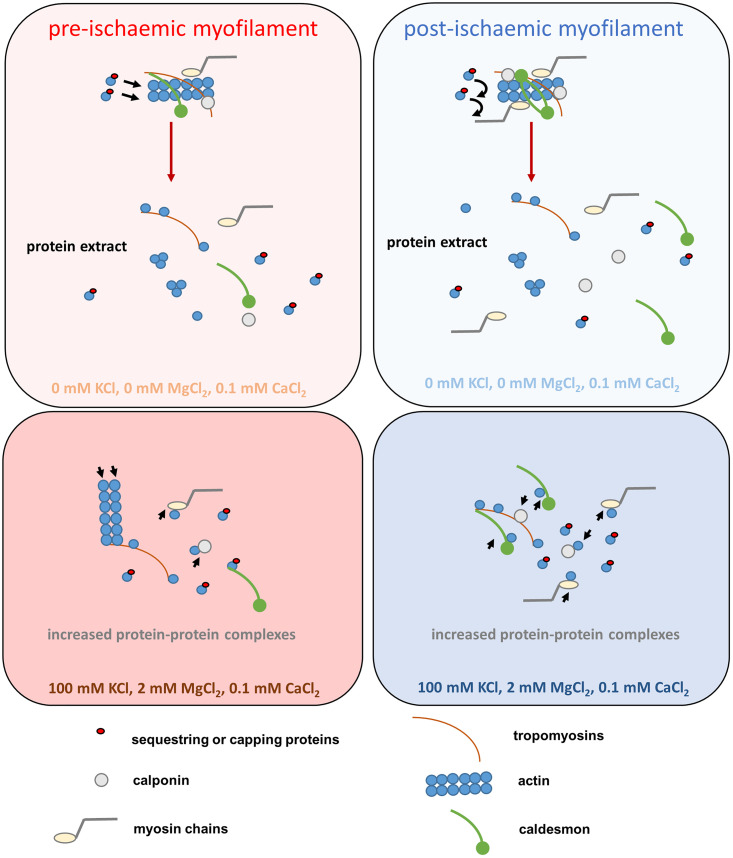
Hypothetical model to describe the molecular changes in cold ischaemic myometrium. The preparation of the protein extracts revealed a remarkable increase in the concentration of ABP proteins such as calponin, myosin and tropomyosin in the PostI samples. We can assume that the proteins formed a large number of complexes with an increased number of molecular interactions under the given high salt conditions. The relatively high number of different ABPs in PostI samples may alter the size and stoichiometry of protein complexes to an increased extent. It is likely that the extracted pool of free actin monomers was reduced by the relatively high number of actin sequestering proteins (e.g., profilin, CapG, gelsolin, etc.) under the high salt conditions in the PostI samples, so that actin nucleation and filament formation did not occur. Therefore, the ABPs removed monomers from the remaining actin nuclei.

However, first and last, protein extracts in the absence of large membrane and cytoplasmic compartments were based on more sustained myofilamental protein complexes [70,81]. We created a hypothetical model to describe the molecular differences of myometrium extracts from PreI and PostI samples (see Fig 5).

Previous studies investigated that the expression of proteins regulating smooth muscle contraction is impaired by ischaemic stress, while the activity of ROCK (Rho-kinase) is stopped and the levels of caldesmon, calponin and MLCK (myosin light chain kinase) were increased [82,83]. In addition, actin capping and sequestering proteins cause cytoskeletal irregularities due to stress of ischaemia [84–86]. It follows that smooth muscle may undergo molecular remodeling of contractile filamental system under ischaemic conditions. Preparation of the protein extracts revealed a remarkable increase in the concentration (S1 Fig) of ABP proteins such as calponin, myosin and tropomyosin in the PostI samples after ultracentrifugation. Their intrinsic tryptophan emission was reduced by salt, suggesting the quenching effect of protein-protein interactions [53]. We can assume that the proteins may have formed a large number of complexes with an increased number of molecular interactions under the given high salt conditions. The relatively high number of different ABPs in PostI samples can alter the size and stoichiometry of protein complexes to an increased extent, and the large variety of complexes can also lead to higher light scattering and thus lower fluorescence anisotropy [54]. Furthermore, under high salt conditions, phalloidin may only bind to short actin oligomers and not to PostI proteins, indicating the ability of ABPs to deplete the ratio of monomers to polymers through the low dissociation of heterologous complexes [55–60]. It is likely that the extracted pool of free actin monomers was reduced by the relatively high number of actin sequestering proteins (e.g., profilin, CapG, gelsolin, etc.) under high salt conditions in the PostI samples, so that actin nucleation and filament formation did not occur. Therefore, the ABPs removed monomers from the remaining actin nuclei so that phalloidin could no longer bind to actin in the absence of actin filaments or nuclei [87–90]. The native dynamics of proteins in PreI samples with intact hydrate envelope may deny eosin binding due to the less positively charged surface [61], so that all possible cation binding sites of the proteins were saturated under high salt conditions and bound more eosin [62]. The PostI extract contains more long protein chains and there are more protein-protein interactions than in the PreI extract, so that eosin preferentially binds to them [63,64].

In our study, we examined the molecular changes in uterine tissue after cold ischaemia using fluorescence spectroscopy in correlation with histological findings. This method has been shown to be effective and sensitive in detecting early structural changes that may affect later function. Further studies with larger sample sizes and longer ischaemic storage times are needed to gain a better understanding and more accurate determination of the tolerance time of the uterine myometrium to cold ischaemia. The clinically most important future study is the investigation of tissue recovery and molecular reconstruction of the contractile filament system after reperfusion.

## Conclusions

In this study, we have shown that cold ischaemic molecular changes in the uterine myometrium can be investigated by fluorescence spectroscopy supported by histological imaging. Our data results indicate that cold ischaemia causes minor changes in myometrial smooth muscle within a clinically relevant period of one hour. Ischaemic stress leads to minor cell destruction with slightly more effective hematoxylin-eosin staining. Myometrial lysate contained large amounts of actin extracted after ischaemic stress with ABPs such as tropomyosins, caldesmons, calponin, and myosin chains. The solubility of actin may also have been provided and protected by capping and sequestering proteins. Fluorescence emission and anisotropy assays with Alexa488-phalloidin, eosin or intrinsic tryptophan proved to be sensitive methods for the detection of structural dynamic changes of myofilament proteins. The results indicate that the contractile filament system underwent molecular stabilization and reassembly by ischaemic stress and that the actin monomers were unable to form polymers due to the increased protein-protein interactions. The content of necrotic proteins cannot be detected after short ischemia, but eosin prefers long proteins (caldesmon, myosin chains, tropomyosin) and a high number of protein-protein interactions under high salt conditions. The results of this study support the feasibility of short-term cold ischemia preservation in uterine transplantation and may highlight the potential for low-risk application in clinical trials for complete myometrial regeneration after reperfusion. However, the study addresses only a short-term ischemic period, and its findings should not be generalized to prolonged storage without future multi-time-point validation.

## Materials and methods

This study was conducted at the Preclinical Center of UPMS. We obtained ethical approval from the Animal Experimentation Scientific Ethics Council (BA02/2000-74/2023). The animals involved in the experiments were treated in accordance with the European Council Directive on the protection of animals used for scientific purposes, for which authorization was obtained from the Animal Ethics Committee (FB-SEMÁB-B/121/2023; FG-SEMÁB-B/122/2023;). The operations were performed in accordance with the principles of surgical antisepsis and asepsis in an operating theatre specially equipped for animals.

### Surgical preparation, anesthesia

Nine multiparous, female Land Sheep Merino ewes (*Ovis aries*) purchased from a registered breeder were used for the operations. The animals were free of clinically recognizable diseases; their body weight was between 36 and 70 kg. To alleviate animals suffering we provided the following efforts. After transport, the sheep were housed in a specially designed, stimulus-flooded pen (floor area between 9.29 and 9.66 m^2^) for 48 hours for acclimatization, followed by 24 hours of food and 12 hours of fluid deprivation prior to surgery. For premedication, the animals were administered diazepam (Seduxen, Richter Gedeon Nyrt., Budapest, Hungary) intramuscularly (0.2–0.3 mg/kg), then, while they were anaesthetized, a peripheral venous cannula (18 G) was placed in the forelimb, through which they also received diazepam intravenously (0.2–0.3 mg/kg). General anesthesia was achieved by administration of thiopental (Thiopental Sandoz, Sandoz Gmbh, Kundl, Austria) (10−20 mg/kg), followed by endotracheal intubation. Narcosis was maintained with sevoflurane (Sevorane, Abbvie PM, North Chicago, Illinois, USA) (3.0–3.3 etSev%). A central venous catheter was placed in the left jugular vein to ensure effective and accessible venous access. During the procedure, the animals’ oxygen saturation, blood pressure and end-tidal carbon dioxide were monitored. The animals were protected from hypothermia with a warming blanket and received a warmed polyionic isotonic crystalloid infusion (NaCl 0.9% Fresenius, Fresenius SE & Co. KGaA, Bad Homburg, Germany) at 10 ml/kg/h for fluid replacement. At the end of the operations, the test animals were sacrificed with a 40% euthanasia solution (Alfasan Nederland B.V., Utrecht, Netherlands).

### Surgical procedure

The surgical plan was based on the method described by Dahm-Kähler et al. [91]. The pelvic anatomy of the sheep, especially the vascular anatomy, shows moderate differences compared to humans. The main abdominal artery divides into three branches (trifurcation), then the main supply artery of the bicornuate uterus (uterus bicornis), the uterine artery, arises on both sides of the internal iliac artery, and the main draining vein, the utero-ovarian vein, opens into the internal iliac vein. After dorso-lateral positioning, antiseptic cleansing and isolation, the abdominal cavity was opened by a total median laparotomy, leaving out the subcutaneous mammary veins. In the abdominal cavity, the four-part stomach was emptied and the intestines were packed in a sterile bag and placed in an extracorporeal position. The pelvis was then exposed with the help of self-retaining and abdominal retractors. The broad ligaments were transected on both sides, then proceeded in the retroperitoneal plane to transect the utero-ovarian vein to its origin on both sides. The ureters were identified and elevated with a rubber sling. The origin of the uterine arteries was dissected and cut in an antegrade direction, paying attention to the ureters. Prior to organ removal and clamping of the vessels, 10,000 IU Na-heparin (Heparinbene Na 25000 NE, Teva Zrt. Debrecen, Hungary) was administered intravenously to prevent clot formation. The vessels were ligated distally of the organ and clamped proximally, whereby the remaining 6–8 cm long vascular pedicles were resected. After exposure of the recto-uterine and vesico-uterine spaces, a hysterectomy was performed. The removed organ was placed on a sterile table for the back-table work. The uterus was resected between the two horns for subsequent procedures. One horn was used to collect various tissue blocks as PreI samples, the other horn was cannulated through the contralateral uterine artery and flushed with constant pressure (5000 IU Na-heparin/500 ml 0.9% NaCl solution) until clear fluid appeared on the venous side. After complete flushing of the blood, the organ was subjected to cold ischaemia, maintaining continuous perfusion with the same solution, and placed in a sterile organ bag at 4 °C ice water for 60 minutes. Perfusion was stopped one hour later.

### Sampling

To follow the recommendations of ARRIVE essential 10 (https://arriveguidelines.org/) we set sampling in hierarchy as removing uterine, obtained tissue blocks, prepared cell lysates then protein extracts. In all 9 (n = 9) successful interventions, two different types of tissue blocks (complete uterine wall and myometrium) were removed at the same time. The PreI samples from the first horn were taken fresh from the uterus immediately after resection. The PostI samples were excised after 1 hour of cold ischaemia. Each uterine block was cut evenly into 30 mg pieces. Blocks were stored in 5 mL Leibovitz medium (Sigma-Aldrich, St. Louis, MO, USA) and then rapidly frozen with liquid nitrogen. We then lysed the tissue blocks by sonication in 30 mL low-salt buffer A (2 mM TRIS.HCl, 0.1 mM CaCl_2_, 0.2 mM ATP, pH7.4). Actin is the most abundant protein in many cells and accounts for about 20% of the total protein content in smooth muscle [47]. Thus, we can achieve a maximum actin concentration of 0.6 mg/ml. The lysates were purified by centrifugation at 2600 xg for 10 min. The protein extracts were obtained by ultracentrifugation of the lysates (100000 xg, 1 h, 4°C). The total protein content of the extracts was normalized to an identical actin concentration (6 μM) by SDS-PAGE analysis and by measuring the total tryptophan absorbance at 280 nm (Jasco V-550 spectrophotometer).

### Histology

To support routine histological examination, samples were fixed in 10% neutral buffered formalin for 24 hours. Tissue blocks were embedded in paraffin, and 2–4 micrometer thick sections were cut and stained with HE dye. HE-stained histological sections of the uterus after warm and cold ischaemia and after implantation were compared with the native samples and analyzed using Case Viewer software (3DHistec Ltd.) and evaluated by two independent pathologists.

### Fluorescence spectroscopies

The smooth muscle extract contains a wide range of proteins with tryptophan residues. The tryptophans were used as intrinsic probes for fluorescence emission-coupled measurements of the structural dynamics of PreI and PostI samples. The structural modification of chains of neighbouring tryptophans can influence their emission spectra. Alexa488-phalloidin (Invitrogen, Thermo Fisher Scientific, Waltham, MA USA) was used at a concentration of 100 nM to measure the amount of filamentous actin polymerisation under high salt conditions (100 mM KCl, 2 mM MgCl2, 0.1 mM CaCl2). The Eosin, EA-50 stain (Abcam Limited, Cambridge, UK) was used at a concentration of 100 μM to analyse the level of necrotic or eosinophilic proteins in the samples. To measure the spectra and time-dependent fluorescence emission with an integration time of 2 s at 280/347 nm for tryptophan, we used a Fluorolog-3 spectrofluorometer (Horiba, Jobin Yvon, Palaiseau, France), typically with 3 nm slits. Steady-state fluorescence anisotropy was determined using an average of 10 points with an integration time of 5 s [43], at a wavelength of 280/347 nm for tryptophan, 488/520 nm for Alexa488-phalloidin and 468/538 nm for eosin.

### Analysis and statistics

Statistical analysis was performed in Origin 2018 (OriginLab Corp. Northampton, MA, USA), which was derived from independent samples. The effects of ischaemia were studied on the myometrial samples from 9 animals, each on 5 tissue blocks. They were visualized with spectra and bar charts. To avoid the possibility of a type I error based on data variance, the significance of the data was tested by ANOVA and Bonferroni tests. Statistically significant differences between groups were defined as p-values < 0.05 and are indicated in the text. The time rate of fluorescence emission was determined by fitting the sigmoidal Boltzmann curve to the measured data in Origin. The rate is calculated from the product of the normalized mass of the proteins in the sample and the time constant from the sigmoidal fit.

## Supporting information

S1 Fig SDS-PAGE of myometrial protein extract. (A) Samples were taken from tissue blocks of sheep uterus pre-ischaemia (PreI) and post-ischaemia (PostI) cases. The tissue blocks were lysed and then extracted under low salt conditions (0 mM KCl, 0 mM MgCl_2_, 0.1 mM CaCl_2_). They were then used for spectroscopic analyses either under high salt conditions (100 mM KCl, 2 mM MgCl_2_, 0.1 mM CaCl_2_). SDS-PAGE (10%) was performed on supernatants after ultracentrifugation (100000 xg, 45 min) of the samples to analyse the soluble protein content under different salt conditions. The main component of the samples was actin. Interestingly, more filamentous actin-binding myofilament proteins were obtained from the PostI samples, e.g., myosin chains, caldesmon, tropomyosins and calponin. (B) Uncropped image of S1 Fig. SDS-PAGE of myometrial protein extract.
(DOCX)
