# Supplementary material for: Myofilamental integrity of the myometrium due to cold ischaemia stress during autotransplantation in the experimental sheep model
Source: PLoS One. 2025 Dec 9;20(12):e0338477. doi: 10.1371/journal.pone.0338477 (PMC12688103; doi:10.1371/journal.pone.0338477)
Supplement: S1 Fig — (A) Samples were taken from tissue blocks of sheep uterus pre-ischaemia (PreI) and post-ischaemia (PostI) cases. The tissue blocks were lysed and then extracted under low salt conditions (0 mM KCl, 0 mM MgCl2, 0.1 mM CaCl2). They were then used for spectroscopic analyses either under high salt conditions (100 mM KCl, 2 mM MgCl2, 0.1 mM CaCl2). SDS-PAGE (10%) was performed on supernatants after ultracentrifugation (100000 xg, 45 min) of the samples to analyse the soluble protein content under different salt conditions. The main component of the samples was actin. Interestingly, more filamentous actin-binding myofilament proteins were obtained from the PostI samples, e.g., myosin chains, caldesmon, tropomyosins and calponin. (B) Uncropped image of S1 Fig. SDS-PAGE of myometrial protein extract. (DOCX) [file pone.0338477.s001.docx]

**Supporting informations**

Myofilamental integrity of the myometrium due to cold ischaemia stress during autotransplantation in the experimental sheep model

Bálint Farkas^1,5^, Katalin Türmer^2^, Martin Rozanovic^3^, Kálmán Kovács^1,5^, József Bódis^1,5^, Gábor Jancsó^4^, Gábor Fazekas^4,5*^, Dávid Szatmári^2^

^1^Department of Obstetrics and Gynecology, University of Pécs, Pécs, Hungary;

^2^Department of Biophysics, Medical School, University of Pécs, Pécs, Hungary;

^3^Department of [Anaesthesiology and Intensive Therapy](https://aok.pte.hu/en/egyseg/310), University of Pécs, Pécs, Hungary;

^4^Department of Vascular Surgery, University of Pécs, Pécs, Hungary;

^5^National Laboratory on Human Reproduction, University of Pécs, Pécs, Hungary;

*****Corresponding author

E-mail: [fazekas.gabor@pte.hu](mailto:fazekas.gabor@pte.hu)

**S1 Fig. SDS-PAGE of myometrial protein extract.** (A) Samples were taken from tissue blocks of sheep uterus pre-ischaemia (PreI) and post-ischaemia (PostI) cases. The tissue blocks were lysed and then extracted under low salt conditions (0 mM KCl, 0 mM MgCl_2_, 0.1 mM CaCl_2_). They were then used for spectroscopic analyses either under high salt conditions (100 mM KCl, 2 mM MgCl_2_, 0.1 mM CaCl_2_). SDS-PAGE (10%) was performed on supernatants after ultracentrifugation (100000 xg, 45 min) of the samples to analyse the soluble protein content under different salt conditions. The main component of the samples was actin. Interestingly, more filamentous actin-binding myofilament proteins were obtained from the PostI samples, e.g. myosin chains, caldesmon, tropomyosins and calponin. (B) Uncropped image of S1 Fig. SDS-PAGE of myometrial protein extract.
